# Through-silicon *via* submount for the CuO/Cu_2_O nanostructured field emission display

**DOI:** 10.1039/c7ra12368j

**Published:** 2018-01-02

**Authors:** Chun-Liang Lu, Shoou-Jinn Chang, Ting-Jen Hsueh

**Affiliations:** Institute of Microelectronics and Department of Electrical Engineering, National Cheng Kung University Tainan 701 Taiwan; National Nano Devices Laboratories Tainan 741 Taiwan tj.Hsueh@gmail.com

## Abstract

A three dimensional (3D) field emission display structure was prepared using CuO/Cu_2_O composite nanowires (NWs) and a three dimensional through silicon *via* (3D-TSV) technique. The experimental results indicated that the diameter and length of the Si *via* were about 100 μm and 200 μm, respectively. For the 3D field emission structure, high-density CuO/Cu_2_O composite nanowires (NWs) were grown on the concave TSV structure using thermal oxidation. The field emission turn-on field and enhancement factor of the CuO/Cu_2_O composite NWs were 15 V μm^−1^ and ∼1748, respectively. With regard to field emission displays, we successfully used the 3D field emission structure to excite the orange phosphors.

## Abstract 2

A three dimensional (3D) field emission display structure was prepared using CuO/Cu_2_O composite nanowires (NWs) and a three dimensional through silicon *via* (3D-TSV) technique.

## Graphical abstract

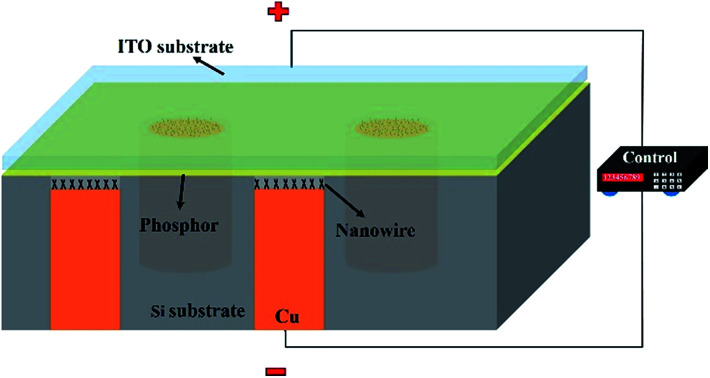

## Introduction

Semiconductor nanowires (NWs) have been investigated for a broad range of potential applications, such as in electronics,^1^ optoelectronics,^2^ field emission,^3^ the biosciences, and energy sciences. p-Type semiconductors with cupric oxide (CuO)^4^ have attracted much attention because of their interesting properties and potential applications in field emission devices, solar cells, superconductors, and photo detectors. Cupric oxide (CuO) and cuprous oxide (Cu_2_O) are p-type semiconductors with monoclinic and cubic crystalline structures, respectively. In the last few years, various CuO nanostructures have drawn research attention for use in applications such as solar cells,^5^ high-temperature superconductors,^6^ gas sensors,^7^ nanorods,^8^ and field emission devices.^9,10^ CuO is a suitable field emitter for such uses due to its rather small bandgap and good conductivity compared to those of other metal oxide materials.^11,12^ NWs are attractive as components of field emitters due to their high emission current density and ease of fabrication.^13–15^ Field emission displays (FED) are flat plane displays that have the advantages of low power-consumption, high brightness, good color rendition, short response time, and wide operating temperature range.^16,17^

In recent years, the development of integrated circuits (IC) has followed Moore's law. However, current semiconductor processing technologies face new challenges when the size of electronic components is downscaled to 10 nm and beyond. 3-D integration using through silicon *via* (TSV) technology is one solution to overcome the scaling limit, and to realize functional diversification for Si-based ICs. TSV technology has been used in microelectromechanical systems (MEMS),^18^ and can enhance the performance of 3D ICs. A lot of effort has thus been devoted to simplifying the TSV process to increase stability and reduce power consumption.

In this work, we integrated the CuO/Cu_2_O composite nanowires (NWs) and the three-dimensional through silicon *via* (3D-TSV) technique to fabricate a 3D field emission structure. CuO/Cu_2_O composite nanowires are grown on a concave TSV structure. The detailed fabrication of the TSV and the electro properties of the fabricated materials are also discussed. These structures are then combined with phosphors to complete a 3D flat display.

## Experimental

**Fig. 1:**
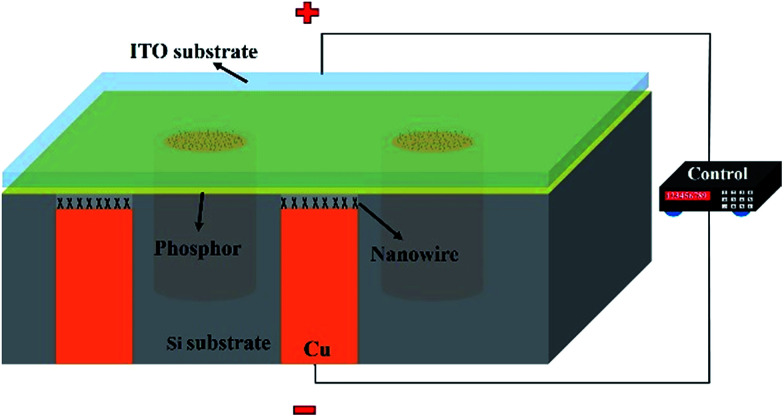
Schematic diagram 3D field emission display.

Fig. 1 shows a schematic of the fabricated 3D field emission display. To fabricate the concave TSV structure, a 6-inch silicon substrate with a (100) orientation was wet cleaned in a standard RCA process. A 300 nm thick Al layer was then deposited by sputtering deposition for use as the etching barrier layer. Standard photolithography was used to make a mask for etching the Al layer. The exposed Al was then wet etched using aluminum etch, after using acetone to remove the positive type photoresist. For the TSV process, the flow of SF_6_ gas, O_2_ gas, substrate temperature, etching time, electrode gap, RF power, and chamber pressure were set at 6 sccm, 50 sccm, −117 °C, 180 min, 7 cm, 1200 W, and 10 mTorr, respectively. The patterning Al layer was then removed by aluminum etch. Next, a SiO_2_ isolation layer with a thickness of 1 μm was formed by thermal oxidation. The Ti adhesion layer was deposited by thermal evaporation with a thickness of 100 nm. The Cu seed layer was also deposited by thermal evaporation, with a thickness of 500 nm. After the Cu electroplating process, a 15 μm thick negative-type PR was spin-coated on the TSV structure, and then standard photolithography was used to define pattern. For the Cu electroplating process, the solution consisted of CuSO_4_, H_2_SO_4_ and suitable organic additives. The plating current and temperature were fixed at 0.1 A and 25 °C, respectively. The backside of the substrate was then polished the backside using chemical–mechanical planarization (CMP). Finally, the wafer was put into H_2_SO_4_ solution to etch the copper and thus achieve a concave structure.

To produce the 3D field emission display, the concave TSV structure was then put into a furnace and heat-treated at 550 °C in ambient air for 6 h to grow CuO/Cu_2_O NWs as an emission structure. For the light layer structure, off-the-shelf orange phosphor was deposited on an indium tin oxide (ITO) electrode. Finally, we integrated the emission structure and light layer structure to form the 3D field emission structure.

The morphology, crystallinity, and optical properties were measured using field-emission scanning electron microscopy (FESEM, JEOL JSM-7000F) and X-ray diffraction (XRD, Rigaku D/MAX2500). The current–voltage (*I*–*V*) and field emission measurements were conducted using a high-voltage source meter (Keithley 4210) under a vacuum.

## Results and discussion

**Fig. 2:**
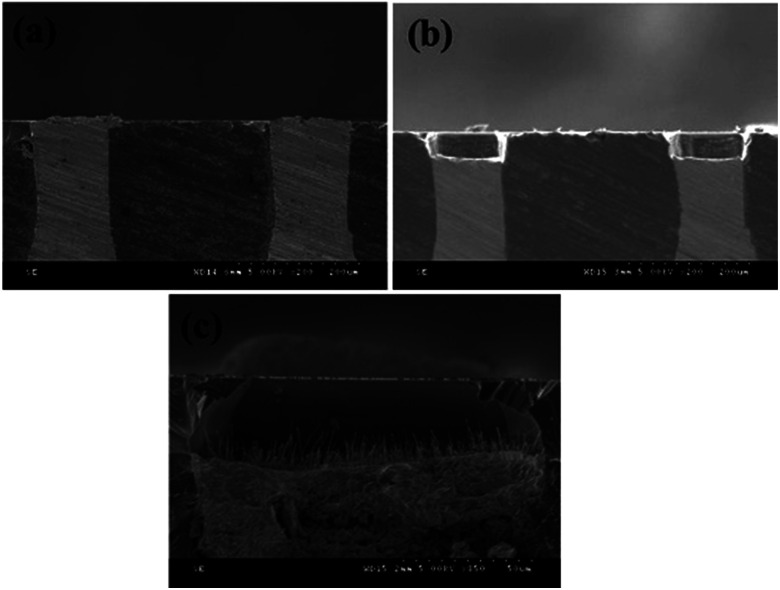
The cross-sectional SEM images of (a) a Cu/TSV, (b) a concave structure, (c) the high-density copper oxide based NWs were grown on the top of the concave structure.

Fig. 2(a) shows a cross-sectional SEM image of a Cu/TSV. The Cu uniformly filled in each TSV. The gaps between the TSVs are about 270 μm. The side length and width of the TSVs were about 240 μm and 120 μm, respectively. To carry out the TSV principle etching mechanism of the cryogenic DRIE, both SF_6_ and O_2_ were provided as a continuous gas flow inside the reactor. During the process these gases react with silicon and form a solid passivation layer of SiO_*x*_F_*y*_ at surface temperatures below −117 °C. Due to directed kinetic energy transfer by the ions, the bottom is by far more easily cracked than the sidewalls. Therefore, the DRIE etch reactions for the TSV structure are as shown in the following equations:^19^

1 SF_6_ + e^−^ → S_*x*_F_*y*_^+^ + S_*x*_F_*y*_˙ + F˙ + 2e^−^

2 O_2_ + e^−^ → O^+^ + O˙ + 2e^−^

Fig. 2(b) shows the cross-sectional SEM image of the Cu TSV plating after etching with a concave structure. It can be seen that the diameter and depth of the concave structure were about 120 μm and 40 μm, respectively. According the past thermal oxidation reports, copper oxide based NWs grow within the temperature range 350–700 °C, and the results of the current study are compatible with previous reports. The atmospheric oxygen reacted with the Cu TSV, the surface of which quickly oxidized to copper oxide based NWs. Fig. 2(c) shows the high-density copper oxide based NWs were grown on the top of the Cu TSV after heat treatment at 550 °C for 6 h. The average diameter and length of the NWs were ∼100 nm and ∼20 μm. The chemical reactions for the CuO composite NWs grown are as follows:

3 2Cu + O_2_ → 2CuO

4 4Cu + O_2_ → 2Cu_2_O

5 2Cu_2_O + O_2_ → 4CuO

**Fig. 3:**
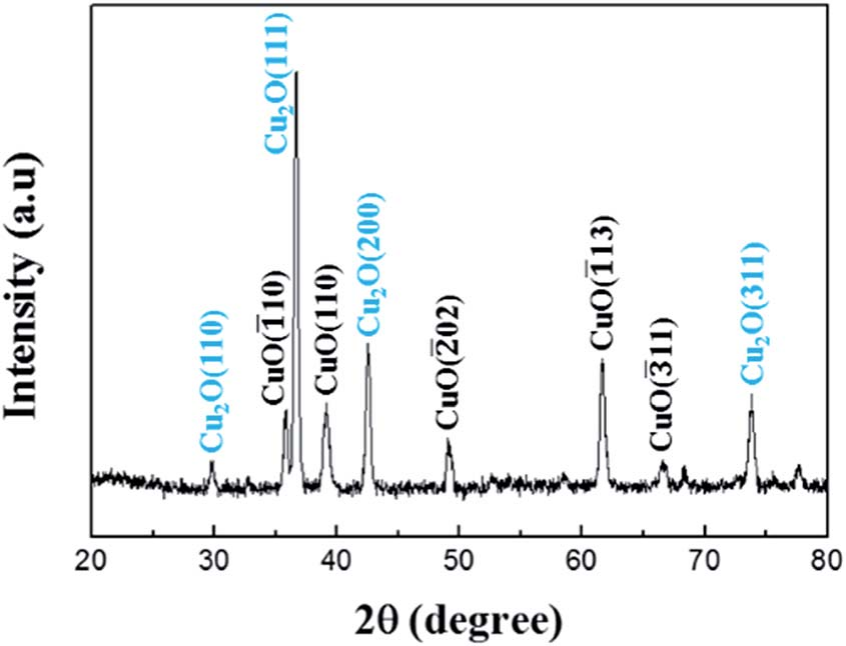
The XRD scan pattern of the copper oxide based NWs.

Fig. 3 shows the XRD scan pattern of the copper oxide based NWs. The XRD peaks demonstrate that the NWs have the CuO monoclinic and Cu_2_O cubic crystalline structures (JCPD card no. 89-2530 and 65-3288, respectively). The Cu_2_O (111) peaks are much more intense than the other peaks.

**Fig. 4:**
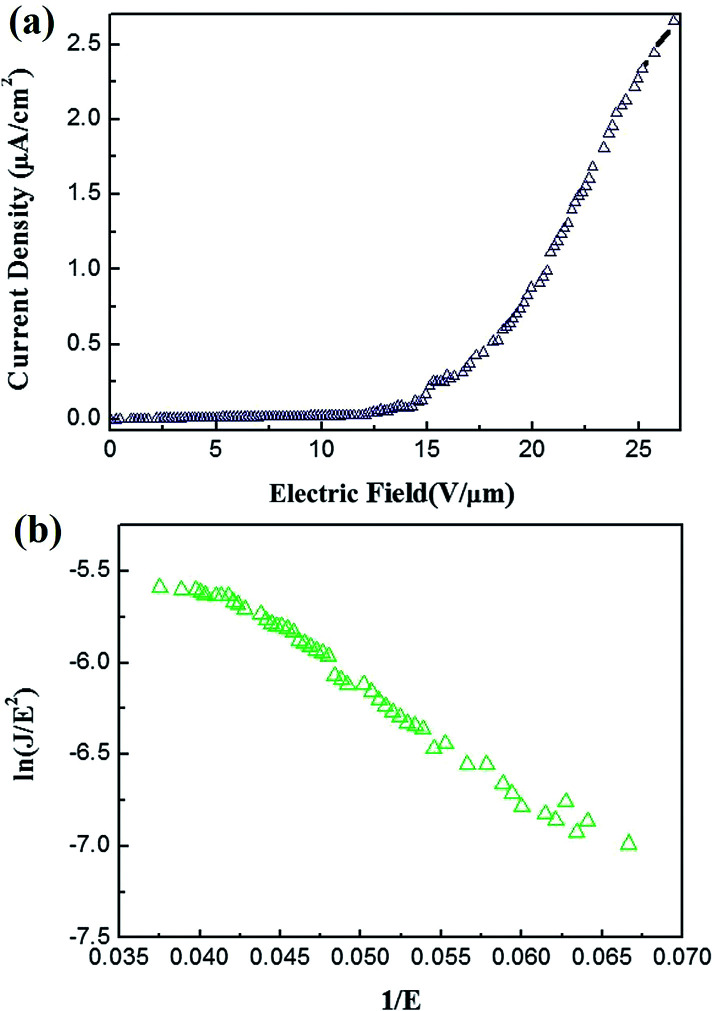
(a) The field emission of the CuO/Cu_2_O composite NWs measured at room temperature in the dark, (b) the F–N plot.

Fig. 4(a) shows the field emission spectra of the CuO/Cu_2_O composite NWs measured at room temperature in the dark. The field emission turn-on field of the CuO/Cu_2_O composite NWs was 14.5 V μm^−1^. The field emissions are described by the Fowler–Nordheim (F–N) equation:^20^6
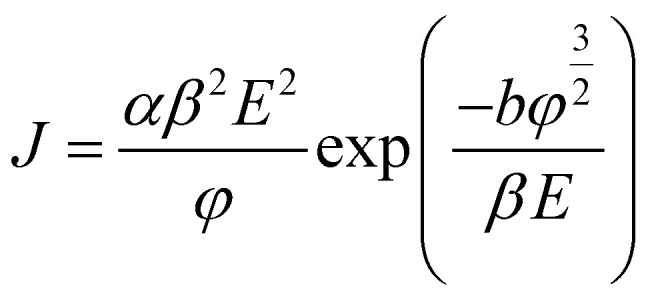
where *J* is the current density (A m^−2^), *a* = 1.54 × 10^−6^ (A eV V^−2^), *b* is the field enhancement factor, *E* is the applied electric field, *f* is the work function (eV), and *b* = 6.83 × 10^−3^ (V μm^−1^ eV^−3/2^). Fig. 4(b) shows the F–N plot. The work function of CuO is 5.31 eV. The enhancement factor *β* is 1748, as calculated from the curve slope.

**Fig. 5:**
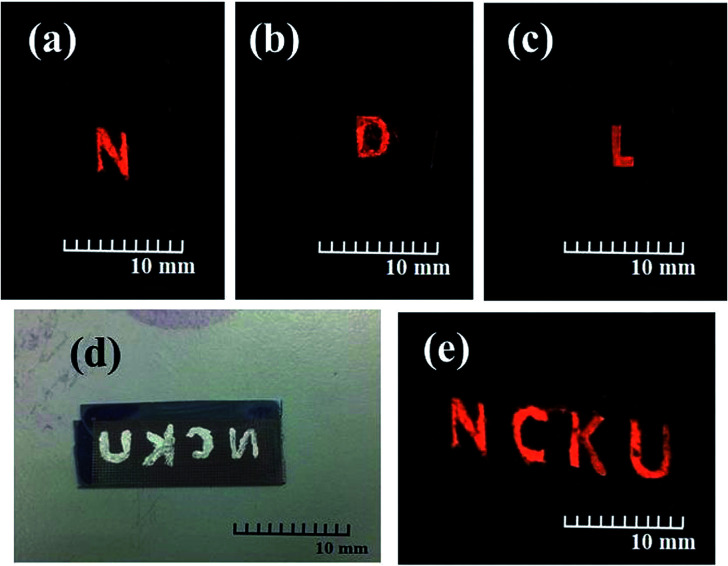
(a)–(c) The photograph of excite orange phosphor, (d) the photograph of the NCKU word prepared on the rear electrodes with the Ag glue, (e) the light layer displayed the NCKU word.

To obtain a 3D nanostructure field emission display, the light layer structure was placed on top of the CuO/Cu_2_O nanostructure field emission structure, and then the 3D nanostructure field emission display was put into a vacuum chamber. Fig. 5(a)–(c) show images of the excited orange phosphor. The rear electrodes (non-nanostructure) were prepared using Ag glue. The emissions conditions are 600 volts under a vacuum of 5 × 10^−6^ Torr. Fig. 5(a)–(c) show the letters N, D, and L on the rear electrodes with the Ag glue, respectively, while Fig. 5(d) shows a photograph of the word NCKU. The emissions conditions are 600 volts under a vacuum of 5 × 10^−6^ Torr. The light layer shows the word NCKU, as shown in Fig. 5(e). As such, it can be concluded that this study successfully fabricated a 3D field emission display using TSV and the CuO/Cu_2_O nanostructure technology. Furthermore, this study showed that we can integrate this with a controller, circuit design, and layout design to easily control the words or images shown.

## Conclusions

In summary, a 3D field emission display structure was prepared in this work using CuO/Cu_2_O composite nanowires and the 3D-TSV technique. With regard to the structure of the 3D-TSV, the diameter and length of the Si *via* were about 100 μm and 200 μm, respectively. For the 3D field emission structure, high-density CuO/Cu_2_O composite nanowires (NWs) were grown on the concave TSV structure using thermal oxidation. The field emission turn-on field and enhancement factor of the CuO/Cu_2_O composite NWs were 15 V μm^−1^ and ∼1748, respectively. We successfully obtained a light layer structure covering the CuO/Cu_2_O nanostructure field emission structure, thus producing a 3D field emission display. Moreover, we found that it is relatively simple to control the positions of the rear electrodes, and thus show the words or images that are wanted.

## Conflicts of interest

There are no conflicts to declare.

## Supplementary Material
